# Non-severe nocturnal hypoglycemic events: experience and impacts on patient functioning and well-being

**DOI:** 10.1007/s11136-012-0234-3

**Published:** 2012-07-24

**Authors:** Meryl Brod, Betsy Pohlman, Michael Wolden, Torsten Christensen

**Affiliations:** 1 The Brod Group, 219 Julia Avenue, Mill Valley, CA 94941 USA; 2 Novo Nordisk A/S, Vandtårnsvej 114, 2860 Soeborg, Denmark

**Keywords:** Nocturnal hypoglycemia, Patient experience, Well-being, Daily functioning

## Abstract

**Purpose:**

Non-severe nocturnal hypoglycemic events (NSNHEs) are hypoglycemic events that occur during sleep but do not require medical assistance from another individual. This study was conducted to better understand the NSNHEs as patients actually experience them in their daily life, and how they impacted functioning and well-being.

**Methods:**

Nine focus groups were held in four countries with diabetics (Type 1 and Type 2) who had experienced an NSNHE within the previous month: France (2 groups); Germany (2 groups); United Kingdom (2 groups); and United States (3 groups). These groups were audio-taped, translated to English where applicable, and analyzed thematically.

**Results:**

Seventy-eight people with diabetes participated in the focus groups: 41 (53 %) were female and 37 (47 %) were male; 24 (31 %) had Type 1 diabetes, and 54 (69 %) had Type 2 diabetes. Participant reports were grouped into several major themes: next day effects, symptoms, sleep impacts, social impacts, corrective action, practical management, feelings about NSNHEs, and work impacts.

**Conclusions:**

People with both Type 1 and Type 2 diabetes experience NSNHEs. The range of impact on these patients is wide, from very mild to severe with a majority of participants experiencing strong impacts that limit their daily functioning. This finding suggests that NSNHEs are more impactful than previously believed.

## Introduction

Hypoglycemia in general, including non-severe nocturnal hypoglycemic events (NSNHEs), is a frequent complication of anti-diabetic medication. NSNHEs are rarely studied for their impacts on patient function or well-being and may be misunderstood and underreported by healthcare practitioners [1]. NSNHEs are hypoglycemic events that occur during sleep but do not require medical assistance from another individual. The patient is able to administer corrective action upon awakening, although they may enlist someone to assist for social support. In a recent survey of 6,756 adults with diabetes (including both Type 1 or Type 2 diabetics), 16.6 % of the sample reported having at least one NSNHE in the previous month [2].

Research on NSNHEs is limited. A review of the literature reveals that the majority of research that has been conducted has focused on either severe hypoglycemic events during sleep, or laboratory-induced NSNHEs. The conclusions drawn from these investigations are not generalizable to the everyday life of patients with diabetes. There is a lack of data specific to how NSNHEs are experienced by patients, and their impacts or consequences. There is evidence of sleep-related hypoglycemia-associated autonomic failure among those with Type 1 diabetes that result in a failure of the patient to awaken during hypoglycemia [3]. These researchers conclude that this failure, coupled with imperfect glucose control, results in a high frequency of NSNHEs. Additionally, studies have found that study subjects are more susceptible to fatigue and impacts on general well-being following NSNHEs [4, 5].

Furthermore, research on the impacts on non-severe hypoglycemic events generally (day or night) concludes that non-severe hypoglycemia is associated with substantial negative economic consequences for both patients and their employers, resulting from lost productivity and missed work time. Productivity loss was highest for NSNHEs that occurred during sleep [2]. Additionally, quality of life decreases as the frequency and severity of hypoglycemic events (day or night) increase [6].

Only a small number of studies have evaluated the impact of NSNHEs on diabetes management, sleep quality, daily functioning, productivity, well-being, and/or quality of life in adults with diabetes [2–7]. In order to bridge this gap in research, this qualitative study was conducted to better understand the NSNHEs as patients actually experience them in their daily life. The concepts underlying this paper are as follows: (1) *patient functioning*, which refers to the patient’s day-to-day ability to accomplish their tasks; (2) *well*-*being*, which refers to their psychological state; and (3) *patient perspective*, which refers to how the patient views their experience (of NSNHEs).

Using focus group interviews conducted in North America and Europe, the primary objective was to qualitatively characterize and describe the experience of NSNHEs and their impacts on patient functioning and well-being.

## Methods

A qualitative design was chosen to meet the study objectives, with semistructured focus group interviews conducted in four countries (US, UK, Germany, and France) to gather data on patient experience of NSNHEs. Focus group interviews are suited to this investigation because they help to identify a “range of experiences and perspectives” as well as provide a forum for both agreement and disagreement between participants, which facilitates insight into the variability of experience and the collection of disconfirming evidence [8–10]. Additionally, four country sites were chosen in order to provide an international perspective on NSNHEs and to help mitigate any potential influences specific to health systems or cultural orientations that might affect the experience of NSNHEs. A purposive sampling strategy was used. Participants were identified by professional market research organizations that recruit for and host focus groups at their or their affiliates’ facilities in each of the respective countries. The market research organizations contacted individuals enrolled in their proprietary databases and prequalified them by telephone using a screening script. Participants received an honorarium for their participation (equivalent to 125.00 USD). Each focus group interview ranged from 8 to 11 participants. Each participant received a copy of the informed consent form, and signed documentation of consent was waived for this study. Additionally, they were asked to complete a questionnaire developed specifically for this study for demographics and basic information about patient experience with diabetes (e.g., their level of control of their diabetes, the number of NSNHEs they experience, etc.). This project was approved by an Institutional Review Board.

To be eligible for the study, participants were required to be over the age of 18, read and speak the native language of the country in which they reside, have a diagnosis of Type 1 or Type 2 diabetes, currently be using insulin, oral medications and/or Byetta (GLP-1) to treat their diabetes, and have experienced an NSNHE within the past 3 months. For the purposes of this study, an NSNHE was defined as having typical hypo symptoms (such as shaking, sweating, hunger, tremor, palpitations, confusion) that may or may not have been confirmed by monitoring blood sugar, not having any of the hypo symptoms but monitoring blood sugar showed that it was too low (≤70 mg/dl), or having symptoms but not having low blood sugar. Participants were excluded from this study if they were using an insulin pump.

All focus groups were moderated by professional focus group leaders, and in the first language of the participants. The focus groups were audio-taped, translated into English where appropriate, and transcribed. The first author, who has extensive professional experience in moderating focus groups with patients, moderated the English-language focus groups in the US and UK. Professional facilitators moderated the focus group interviews in France and Germany. The first author coached these moderators and observed these groups with the aid of simultaneous translation to insure quality of discussion. The focus group interviews were semistructured, and the interview guide was designed to elicit participant experience with NSNHEs, their corrective action strategies, and the social and practical impacts of these events on their lives.

The transcripts were analyzed thematically using qualitative analysis software ATLAS.ti, which was chosen for its ability to handle and organize large data sets, provide documentation of the coding process, and assist in organizing disconfirming evidence within the data set [11]. Descriptive coding was used to identify emerging concepts, coded in the chronological order in which the focus groups occurred. These codes were then aggregated into major themes, also used in the Results section of this paper. This coding method is consistent with grounded theory and other thematic analysis methodologies and is well-suited to research on patient experiences [12, 13]. Each transcript was skimmed once, coded, and reviewed. All transcripts were reevaluated for new subthemes that emerged in subsequent transcripts. The first two authors worked together on the analysis, with the first contributing her experience of conducting or attending all of the focus group interviews and the second working primarily with the transcripts. Data were triangulated between the literature, and each of the two authors’ interpretations of patient statements. Thematic saturation, defined as that point in time of the study where no new themes or subthemes emerged, occurred by the 7th focus group that was conducted. Data from subsequent focus group interviews enriched the analysis with their added descriptions of preexisting themes.

## Results

### Sample characteristics

Nine focus groups were conducted in the four countries (cities included New York, Atlanta, Los Angeles, London, Paris, and Frankfurt), totaling 78 participants (Table 1). Forty-one (53 %) participants were female and 37 (47 %) participants were male. Twenty-four (31 %) had Type 1 diabetes and 54 (69 %) had Type 2 diabetes. The mean age of participants was 46.7 years (range: 20–65 years of age). Type I participants were predominantly males (66.6 % of the Type I group), while Type II participants were predominantly females (61.1 % of the Type II group).

**Table 1:** Sample description (n = 78)

	Type I	Type II	Total
	(*n* = 24)	(*n* = 54)	(*N* = 78)
	30.8 %	69.2 %	
Gender; # (%)			
Female	8 (33.3)	33 (61.1)	41 (52.6)
Male	16 (66.6)	21 (38.9)	37 (47.4)
Marital Status; # (%)			
Single	9 (37.5)	15 (27.8)	24 (30.8)
Married	11 (45.8)	22 (40.7)	33 (42.3)
Partnered	2 (8.3)	10 (18.5)	12 (15.4)
Divorced	1 (4.2)	3 (5.6)	4 (5.1)
Widowed	1 (4.2)	4 (7.4)	5 (6.4)
Ethnicity; # (%)			
White/White British/Caucasian	19 (79.2)	38 (70.4)	57 (73.1)
Black/African/African–American	4 (16.7)	7 (13.0)	11 (14.1)
Latino/Hispanic/Mexican–American	0 (0.0)	2 (3.7)	2 (2.6)
Asian	0 (0.0)	2 (3.7)	2 (2.6)
Mixed Race	0 (0.0)	3 (5.6)	3 (3.8)
Other	1 (4.2)	2 (3.7)	3 (3.8)
Age; mean (range)	40.4 (20–59)	50.0 (27–65)	46.7 (20–65)
Work Status; # (%)			
Work full time for pay	16 (66.7)	33 (61.1)	49 (62.8)
Work part time for pay	2 (8.3)	10 (18.5)	12 (15.4)
Not working	5 (20.8)	10 (18.5)	15 (19.2)
Student	1 (4.2)	1 (1.9)	2 (2.6)
Education; # (%)			
Grade school or less	1 (4.2)	1 (1.9)	2 (2.6)
High school or technical school	4 (16.7)	20 (37.0)	24 (30.8)
College	15 (62.5)	24 (44.4)	39 (50.0)
Graduate or professional school	4 (16.7)	9 (16.7)	13 (16.7)
Income^a^; # (%)			
Less than $40,000	9 (37.5)	16 (29.6)	25 (32.1)
$40,000–$60,000	5 (20.8)	15 (27.8)	20 (25.6)
Over $60,000	9 (37.5)	22 (40.7)	31 (39.7)
Blank response^a^	1 (4.2)	1 (1.9)	2 (2.6)
Age when diagnosed; mean (range)	20.7 (1–46)	41.0 (17–62)	34.8 (1–62)
Length of time w/diabetes in years; mean (range)	19.7 (3–44)	8.5 (1–37)	12.0 (1–44)
NSNHE, times per month; mean (range)	4.4 (1–22.5)	3.8 (1–12.5)	4.0 (1–22.5)
		(*n* = 53)	(*n* = 77)
How well diabetes controlled; # (%)			
Very well	1 (4.2)	3 (5.6)	4 (5.1)
Well	12 (50.0)	24 (44.4)	36 (46.2)
Moderately	9 (37.5)	25 (46.3)	34 (43.6)
Poorly	1 (4.2)	0 (0.0)	1 (1.3)
Very poorly	0 (0.0)	1 (1.9)	1 (1.3)
Blank response^a^	1 (4.2)	1 (1.9)	2 (2.6)

^a^ *Blank response* the participant did not fill in this question

The average duration of diabetes for the sample was 12 years (range: 1–44 years). Over half of the sample considered their diabetes to be controlled well or very well (51.3 %), and 34 participants (43.6 %) considered their diabetes to be controlled moderately well. Both Type 1 and Type 2 diabetics reported that they experienced NSNHEs: Type 1 diabetics reported an average of 4.4 NSNHEs per month (range: 1–22.5 per month) and Type 2 diabetics reported an average of 3.8 NSNHEs per month (range: 1–12.5). The average incident rate of NSNHEs for both groups combined was 4.0 per month (range: 1–22.5).

Ethnically and racially, nearly three quarters of the participants in this study self-identified as White/Caucasian (57 or 73.1 %). A majority of participants in this study worked fulltime (62.8 %), and income was distributed across all income categories.

### Themes generated by focus groups

#### Experiencing NSNHEs

Both Type 1 and Type 2 diabetics experience NSNHEs. Participants noted wide ranges of symptoms and severity of NSNHEs. NSNHEs involved a constellation of symptoms along a continuum ranging from unpleasant to traumatic, with the most common symptom as sweating:

Samantha: What I wake up to is my hair is really, really sweaty. My hands are dripping and my night clothes are absolutely drenched and as soon as you get up you have that chill. (London #1)

Participants noted anxiety or panic, shaking, and confusion or disorientation. Here are three examples of impacts on emotional states described by patients as typical symptoms during an NSNHE:

Anxiety or panic

Florence: I think it’s also kind of like… for me, anyway, it’s sort of like… it’s a shock and like yourself, I kind of get the panic attack as well and then I’ve got to calm myself down from that. (London #2)

Shaking

Male Speaker: I don’t have any palpitations, I am very weak and shaking and I feel anxiety; I feel oppressed because it is a situation that is outside your control; each time we hope it’s going to be ok by taking some sugary things, and each time is a surprise. (Paris #2)

Confusion and disorientation

Devon: But the hardest part I find is that when I do get up I’m so confused and I struggle with my speech at times and all sorts, just to get thoughts together and it leaves me drained for a day or two and then it’s very hard to get back to normal life after an incident. (London #1)

Additional symptoms included feeling hot or cold, dizziness, heart pounding or palpitations, restlessness, trouble with walking or balance, bad or weird dreams, vision disturbances, headache, weakness, muscle tension, neuropathy, anger, and nausea. The wide range of symptoms reported suggests that the experience of nocturnal hypoglycemia is individualized.

When participants experienced NSNHEs, their corrective action did not significantly differ from typical corrective action taken for daytime hypoglycemic events. Most participants reported eating or drinking something sweet to raise their blood sugars. Although many participants noted that they check their blood sugar to confirm hypoglycemia, some do not and respond instead to their symptoms alone. Many participants reported that they kept treatment food and drink on their bedside table so that it was nearby in the event of an NSNHE.

Generally, participants reported having strong feelings as a consequence of NSNHEs, and they emphasized fear and worry the most:

Male Speaker: It’s an interruption during the night, it’s a decrease in the level, it’s a fear, a fear of something that I don’t understand and cannot control. (Paris #1)

Additional emotional consequences of NSNHEs included frustration, anger, helplessness, and feelings of sadness.

Participants often compared and reported that the experience of nocturnal hypoglycemia was different from daytime hypoglycemia. They offered varied responses to the differences between daytime and nighttime events; however, the sentiment that NSNHEs are in some way more difficult than daytime events was more common.

#### Sleep Impacts of NSNHEs

Although a few participants suspected that they do not always awaken during an NSNHE, most participants stated that nocturnal hypoglycemia caused them to wake up and take corrective action.

The sleep impact data revealed a wide range of experiences. Some individuals reported that it was easy for them to get back to sleep. However, others reported that it is very difficult to get back to sleep. Participants reported that sleep was very generally disrupted:

Stephanie: I feel that way every night. Well, not that I’m scared every night, but if I feel uncomfortable and can’t fall asleep, then I’m worrying about it. (Atlanta)

Some people remained awake for the remainder of the night. Of those individuals who reported an estimate of the time they spent awake, the range was 5 min to 5 h. Individuals also varied greatly in their tolerance to sleep disruption.

Generally, participants reported that NSNHEs disrupt sleep, which amplifies the next day effects of the event. There was a range of experience with regard to the severity of the sleep disruption, from mild to severe.

#### Next day(s) effects of NSNHEs

The overall experience of the NSNHE was two-pronged, leading to many discomforts the day following the event. These discomforts the next day were the most frequent major theme noted by participants. These effects were caused by: (1) sleep disruption (and resulting sleep deprivation for some), in confluence with (2) physiological effects of fluctuating blood sugars, in the night and throughout the next day. Participants identified these impacts and consequences as disruptive and often upsetting:

Carol: Yes, I feel low, very tired. Very tired. And when I’m at work, I’m just counting down the hours to go home. (London #2)

Khalid: Yes. It’s pretty much like having a really bad hangover. It’s like you’ve gone out for a real session for a drink one night, the next day you feel like tired… that’s just how you feel, it’s like a really bad hangover. (London #2)

Participants reported a wide range of consequences that included feeling irritable, needing naps the next day, and an inability to focus or concentrate the next day:

Irritation

Djamila: Irritation because that’s another night that is broken up into 2 or 3 bits. I won’t feel very fresh in the morning to go to work, so the irritation is more for the next day because it won’t be very good. (Paris #2)

Needing naps

Richard: I’m fine once I’ve had my jam sandwich and tea. The way I get round the tiredness bit is I have catnaps. On the way to work, on the train, I might doze off for about 10 min. On the way back home I might doze off. (London #1)

Inability to focus or concentrate

Wildi: But sometimes I simply cannot comprehend it, that I can’t concentrate, I think to myself, “It cannot be that I’m not able to pull myself together for 5 min.” (Frankfurt #2)

There were only a very few participants who stated that they did not feel particularly tired the following day, and these participants also reported that they were not awake for very long during an NSNHE. Likewise, there were a few incidences of participant reports that they felt fine the next day, experiencing little impact from the event. This demonstrates (again) the individuality and range of the experience of NSNHEs.

However, despite this disconfirming evidence, nearly all commentary about next day effects of NSNHEs was negative. Other effects reported include lowered energy, headaches, the need to sleep in late, awakening to high blood sugars, moodiness, feeling down, nausea, anxiety, and dizziness. In summary, NSNHEs cause a wide range of negative consequences the day following the event that include physiological symptoms, temporary cognitive impacts, and emotional reactions.

#### Social impacts of NSNHEs

The most important consequence reported by participants in all focus groups was that the NSNHE disturbed the sleep of their bed partners, resulting in fatigue for them the following day. All of these reports were of waking bed partners during the night and as a result of an NSNHE:

Neil: But if it seems like more of a minor one but I’m tossing and turning-waking up, even though sometimes I feel like she’s sleeping through it, she’ll say she’s up and she’ll say she’s more tired than me. And she’ll give me a guilt trip over it. “You could’ve been quieter when you got up.” So she does care, but at the same time, she’ll act like she’s more tired than even me. It’s almost a guilt trip thing. (Los Angeles)

Participants also reported that they make an effort to avoid waking bed partners. Here is an example:

Farma: He panics. So I tend to try not to wake him up or let him know what’s going on actually. He hates that but that’s from over the years seeing how he reacts. (New York)

Participants reported that they receive social support from others who are knowledgeable about nocturnal hypoglycemia, especially partners. Support in this context refers to understanding, kindness, and general accommodation for their NSNHE difficulties: social support. However, they also reported that the day after an NSNHE, they have reduced social interaction due to their fatigue levels and general feelings of illness, and described their own social withdrawal due to the event. More specifically, participants stated that they cancel social events due to how they feel after an NSNHE:

Respondent: I just wanted to say, when I’m not feeling well I’m taking a different way home then I go usually, I don’t like going through a particular street, don’t ask me why, I don’t know it, but I don’t like to go into a crowd of people *when I don’t feel well*, so I prefer to go a longer way. And normally I’m very spontaneous and if I get asked to go out after work, then no, I can’t. […] it’s not because I don’t want to see the person, I just don’t want to have company. (Frankfurt #2)

Participants noted that their families were negatively impacted by worry for the participant. Participants’ fatigue also affected their interactions. Participants also noted that sometimes they worried about their family:

Barbara: Because usually you spend time with your kids, the homework, whatever we do, it’s a routine. Sometimes I’m just too tired to even ask them how their day was. I just come in and go straight to bed. (New York)

Some participants regularly received assistance from others in their households, even though they did not require this assistance:

Victoria: I normally… I’m normally aware and I just take myself, get myself a drink, but sometimes I’ll have to because I panic from it. And I’ll wake him, he’s great, he’ll go and get me a drink instead. (London #2)

In summary, the strongest social impacts included waking others up during an NSNHE and disrupting their sleep, enlisting other’s support and assistance during the event, and the experience of reduced social interaction the following day by withdrawing or canceling events.

#### Work impacts of NSNHEs

Impacts of NSNHEs on work mirror concerns reported on next day effects of NSNHEs. Broadly, the tiredness and fatigue from sleep disruption and the physiological effects of fluctuating blood sugars contribute to many difficulties at work. The most frequent impacts reported were problems with focus or concentration for work tasks, and reduced productivity while at work. Additionally, participants reported that they call in sick, take extra breaks or leave early, or go to work late in the morning. In summary, NSNHEs have negative work impacts and lead to lost work time.

**Fig. 1:**
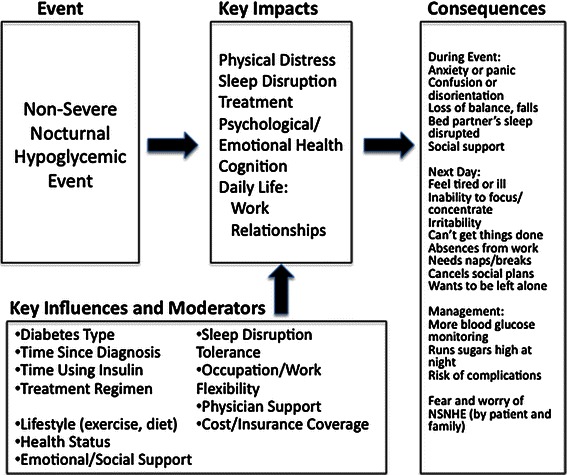
Preliminary Theoretical Model

Based on these findings from the qualitative data, a preliminary theoretical model of the impact of NSNHEs was developed, as shown in Fig. 1.

## Discussion

NSNHEs were found to be disruptive and cause considerable distress to the majority of both Type 1 and Type 2 participants in this study. Among Type 2 diabetics, NSNHEs are not limited to those who are insulin-dependent. These data also suggest that the range of individual experience of NSNHEs is very wide. However, for the majority of participants, the continuum of experience ranges from unpleasant to traumatic. The impacts on the individual’s life and daily routine are equally wide-ranging, from moderate to extreme. This is an important finding as primary care clinicians attending to patients with Type 2 diabetes may not appreciate the severity of the impacts and potential consequences that NSNHEs have on their patient’s functioning and well-being [1].

NSNHEs produce symptoms that awaken and frighten people who experience them and sleep is often disrupted. The day after the event is often a difficult day due to the physiological effects of fluctuating blood sugars (during the event and the following day) and sleep disruption or deprivation. Next day effects are so uncomfortable that they may be more disturbing to individuals than the hypoglycemic event itself. NSNHEs are often thought to be more difficult to predict, understand, or manage as compared to daytime hypoglycemia, and patients are often afraid or worry about their occurrence. Given the challenges of the symptoms and the emotionally laden experience of NSNHEs, participants wanted to avoid them if possible. Some participants stated that they run their sugars high before bedtime as a strategy for avoiding NSNHEs. It is unclear whether this is on advice from their physicians or diabetes educators. Potentially, keeping blood sugar high can lead to other complications of diabetes over time.

Although not a major theme in this data set, a few participants reported that they were prone to falling during their event due to dizziness and disorientation during making this an interesting topic for further inquiry. Furthermore, NSNHEs also have not inconsequential work impacts of reduced productivity and lost work time for both the person with diabetes as well as for some of their bed partners. This echoes the conclusions drawn in a prior study of the impacts of non-severe hypoglycemia, which noted that productivity loss was highest for NSNHEs [2].

It is not surprising that participants recruited for their experiences with NSNHEs would report that NSNHEs are problematic for them, and findings of this study must be placed within this context. However, previous research has found that approximately 16 % of all persons with diabetes experience at least one of these events in the previous month. For this not insignificant percent of persons with diabetes, this study does demonstrate that there are serious negative impacts and consequences that are generally unacknowledged in the literature. Further research is needed in this area in order to evaluate the level of concern this poses for people with diabetes in general. Additionally, the study found that NSNHEs are not only problematic for those using insulin or only those with Type 1 diabetes.

As participants with Type 1 or Type 2 diabetes and insulin and non-insulin users participated in the focus group interviews together, it was not possible to differentiate the experiences of NSNHEs between these two forms of diabetes or treatments conclusively. It is possible that the frequency, severity, and personal reactions may differ between Type 1 and Type 2 diabetics or treatments. In these focus group interviews, there suggestion that participants who had Type 1 diabetes and, therefore, typically had a longer tenure with insulin use and its titration were slightly less bothered by NSNHEs due to familiarity with them. For these individuals, the NSNHE experience entailed similar impacts and consequences physically, but it is possible they were less worried or fearful of them. Future research in this area may illuminate diabetes type-specific moderators for the overall impacts of NSNHEs.

Study limitations include translation and transcription problems for those focus group interviews held in a language other than English as it was difficult for the translators to relate nuances and the full intent of the focus group discussion. This resulted in less description than would occur within the first language. Despite these discrepancies, the data yielded insights that were quite rich in understanding the impact of NSNHEs on these participants.

Additional qualitative and quantitative research is needed to understand the phenomenon of NSNHEs from a physiological, emotional, and social perspective. These data indicate that NSNHEs are not always experienced in the same way as hypoglycemia occurring during the day. Research that explicitly investigates NSNHEs may alter diabetes management strategies overall and lead to new recommendations for the practical and daily management of hypoglycemia.

Finally, this study did not explore the experiences of patients with their healthcare providers about NSNHEs in depth. However, a few participants suggested that physicians may not appreciate the impacts of NSNHEs on patients. In contrast, some participants reported discussing NSNHEs with their doctor and receiving helpful advice. Given the role of physicians and other clinicians in directing diabetes management, it is important to know what influence they may be having on the management and treatment of NSNHEs. Given the substantial negative impacts reported in this study, it would be of concern if health care providers as well as patients do not fully understand these NSNHE impacts. The study findings warrant further research on physicians’ views on NSNHE impacts.

## Abbreviations

NSNHEs: Non-severe nocturnal hypoglycemic events
